# Genomic analysis reveals the presence of a class D beta-lactamase with broad substrate specificity in animal bite associated *Capnocytophaga* species

**DOI:** 10.1007/s10096-016-2842-2

**Published:** 2016-12-01

**Authors:** S. Zangenah, A. F. Andersson, V. Özenci, P. Bergman

**Affiliations:** 1 Department of Laboratory Medicine, Division of Clinical Microbiology, Karolinska Institutet and Karolinska University Hospital, Huddinge, Sweden; 2 Science for Life Laboratory, School of Biotechnology, Division of Gene Technology, KTH Royal Institute of Technology, Stockholm, Sweden

## Abstract

*Capnocytophga canimorsus* and *Capnocytophga cynodegmi* can be transmitted from cats and dogs to humans, and can cause a wide range of infections including wound infections, sepsis, or endocarditis. We and others recently discovered two new *Capnocytophaga* species, *C. canis* and *C. stomatis*, mainly associated with wound infections. The first-line treatment of animal bite related infections is penicillin, and in case of allergy, doxycycline and trimethoprim/sulfamethoxazole. However, there is a lack of antibiotic susceptibility patterns for animal bite associated *Capnocytophaga* species. Thus, we ﻿set out to study the antibiotic profiles against animal bite associated *Capnocytophaga* species isolated from wound and blood cultures after cat and dog bites and coupled the findings to whole genome sequencing data. A total of 24 strains were included in the study. Phenotypic analysis of antibiotic resistance was performed with E-tests. The web-based tool ‘Resfinder’ was used to identify resistance genes in the whole genome dataset. Two strains of *C. cynodegmi* and two strains of the recently discovered *C. stomatis* were resistant to penicillin (MIC > 24 mg﻿/L) and cephalosporins (MIC > 24 mg/﻿L), and three out of these strains also exhibited resistance to imipenem (MIC = 32 mg/﻿L). Genomic analysis revealed that these strains carried a class D beta-lactamase gene, which has not previously been found in *Capnocytophaga* spp. A class D beta lactamase with broad substrate specificity was found in animal bite associated *Capnocytophaga* species, which could have important implications when treating wound infections after cat and dog bites. It also suggests that pet animal bacteria can harbour resistance genes with relevance for human infections.

## Introduction

*Capnocytophaga canimorsus* and *Capnocytophaga cynodegmi* normally reside in the oral flora of healthy cats and dogs, but can be transmitted to humans via bites or scratches. *C. canimorsus* can cause wound infections, but also invasive infections, including sepsis, meningitis, or endocarditis [4], whereas *C. cynodegmi* has mostly been associated with wound infections [9, 13, 23]. Recently, an additional *Capnocytophaga* species named *Capnocytophaga canis* was found in the oral flora of healthy dogs [22]. In addition, we also recently described a fourth animal-associated *Capnocytophaga* species that was given the name *Capnocytophaga stomatis* [26]. The pathogenic potential of *C. canis* is unknown, whereas *C. stomatis* was found in a wound specimen from a dog bite [26].

The treatment of wound infections after cat- and dog-bites involves cleaning and debridation and also antibiotics as prevention or treatment, unless the wound is superficial. For dog-bites, the first-line treatment is amoxicillin+clavulanic acid, and for cat-bites the first-line therapy is penicillin [12]. If the patient is allergic to penicillin, the recommendation is trimethoprim–sulfametoxazol or doxycycline [21]. Clindamycin or daily injections with ceftriaxone—in cases of low compliance—have been proposed [15]. These antibiotics are considered to cover the most common bacteria related to cat and dog bites, including *Pasteurella*, *Staphylococcus*, *Streptococcus* as well as anaerobic *Fusobacteria* spp. and *Bacteroides fragilis*. In addition, the suggested empirical treatment is thought to cover for *C. canimorsus* and *C. cynodemi* [20].

However, available data on antibiotic susceptibility testing (AST) is scarce for *C. canimorsus* and *C. cynodegmi*. The related human *Capnocytophaga* spp., which are found in the oral flora of healthy individuals, are usually susceptible to penicillin. However, penicillin resistance also occurs in these bacteria, and some reports have described beta-lactamase production among clinical isolates of human *Capnocytophaga* spp., including *C. sputigena* and *C. ochracea* [10, 17]. However, whether animal-associated *Capnocytophaga* species produce beta-lactamases is not known.

Thus, given the lack of available AST data, we set out to describe the phenotypic susceptibility patterns for the strains in our collection to a range of clinically relevant antibiotics. Our strain collection included *C. canimorsus* and *C. cynodegmi* as well as the novel *Capnocytophaga* species *C. canis* and *C. stomatis*. In order to obtain a mechanistic understanding of the phenotypic results, we coupled the AST data to bioinformatic analysis of whole genome sequences.

## Material and methods

### Bacterial strains

In total, 24 isolates were included in the study, including *n* = 9 *C. canimorsus*, *n* = 9 *C. cynodegmi*, *n* = 3 *C. stomatis* and *n* = 1 *C. canis*, and *n* = 2 reference strains (*C. canimorsus*, ATCC 35978 and *C. cynodegmi*, ATCC 49045). *C. canis* was isolated from a human wound specimen, and is thus here defined as a clinical isolate. These isolates have been described in detail in previous studies from our group [26–28]. The genomes for all 24 strains were used in the bioinformatic analyses, whereas 16 strains were subjected to antibiotic susceptibility testing based on consistent and reproducible growth patterns.

### Reagents

Horse blood agar plates (HBA) and phosphate buffered saline (PBS) were manufactured by the Substrate Unit at KarolinskaUniversity Hospital, Stockholm, Sweden, which is accredited by the Swedish Board for Accreditation and Conformity Assessment (SWEDAC).

### Antibiotic susceptibility testing by E-test

The Etest (Biomerieux) was used to determine the minimal inhibitory concentration (MIC) against a range of clinically relevant antibiotics: cefotaxime, gentamicin, oxacillin, ceftazidime, imipenem, clindamycin, doxycycline, tigecycline, erythromycin, benzyl penicillin (PcG) and amoxicillin–clavulanate (amox/clav). Briefly, bacteria were thawed from stocks and sub-cultured on HBA plates, incubated at 35 °C in 5% CO_2_ for 48 hours. Thereafter, a loop of bacteria was suspended in sterile PBS to an optical density of McFarland 0.5 × 10^8^ CFU/ml [18], and spread on blood agar plates immediately prior to the application of the E-test strips [11]. Plates were incubated at 35 °C in 5% CO_2_ for 48 hours before reading the results. MIC values were interpreted according to the EUCAST antimicrobial guidelines for non-species-related breakpoints [19].

### Identification of antibiotic resistance genes

The software tool ResFinder from the Center for Genomic Epidemiology was used for identification of antimicrobial resistance genes in the genomic sequences of 24 strains of *Capnocytophaga* spp. FASTA files of complete genomes for all strains were used as search strings in the program. The threshold for ID was set to 98% and the minimum length was set to 60% [29].

### Phylogenetic analysis

The phylogenetic tree of the various beta-lactamases was constructed utilizing the web-based tool found at: http://blac.bii.a-star.edu.sg/.

## Results

### Antimicrobial susceptibility testing

Given the lack of knowledge on antibiotic resistance in animal bite associated *Capnocytophaga* species, we determined the minimal inhibitory concentrations (MIC) of the strains in our collection (*n* = 16) (Table 1). Importantly, all *C. canimorsus* strains, obtained from blood cultures, were susceptible to penicillin (MIC 0.025–2 mg/l), which is the recommended first choice in relation to dog and cat bite wounds [12]. Likewise, the *C. canimorsus* strains were susceptible to cefotaxime and ceftazidime as well as to imipenem, which all are common clinical choices in case of sepsis with unknown cause. We also included several strains that were isolated from wounds. Interestingly, four wound-associated strains (W5, W6, W7, and W10) were resistant to penicillin and cephalosporins, and three of these strains also exhibited a high MIC value for imipenem, suggesting the presence of a beta-lactamase with broad substrate specificity. Notably, these four strains, of which two belonged to *C. cynodegmi* and two belong to the recently described *C. stomatis*, were also resistant to amoxicillin+clavulanic acid and negative for cefinase activity (Table 1).

**Table 1:**
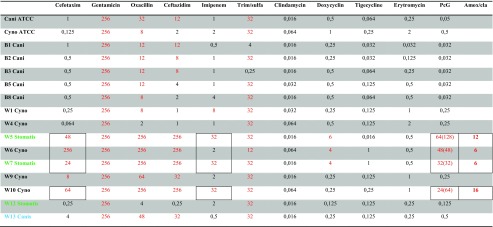
Phenotypic antibiotic susceptibility results for *C. canimorsus* (Cani), *C.cynodegmi* (Cyno), *C. canis* and *C. cynodegmi*. Red colour denotes resistant phenotypes. Squares marks isolates with confirmed genomic presence of blaOXA-347. B, isolates obtained from blood cultures; W, isolates obtained from wound cultures

### Searching for antibiotic resistance genes using whole genome data

Since we previously performed whole genome sequencing of these strains, we set out to search for antibiotic resistance genes that could explain the phenotypic results for strains W5, W6, W7, and W10. To this end, we used the web-based program ResFinder, which demonstrated the presence of a class D beta-lactamase in two strains of *C. cynodegmi* and two strains of *C. stomatis* (Table 2). The gene was designated ‘blaOXA-347’ and showed 100% identity between the four strains, indicating a common origin. In addition, the tetracycline resistance gene ‘tet(X)’ was found in W6 and W7, which was in line with the phenotypic data in Table 1.

**Table 2:** Results for identified resistance genes in *C. cynodegmi* and *C. stomatis* by Resfinder

Bacteria ID	Resistance gene	% identity	Query/HSP length	Predicted phenotype
W5 C. stomatis	*blaOXA-347*	100.00	825/825	Beta-lactam resistance
W6 C. cynodegmi	*blaOXA-347*	100.00	825/825	Beta-lactam resistance
	*tet(X)*	89.55	1,167/1,167	Tetracycline resistance
W7 C. stomatis	*blaOXA-347*	100.00	825/825	Beta-lactam resistance
	*tet(X)*	89.55	1,167/1,167	Tetracycline resistance
W10 C. cynodegmi	*blaOXA-347*	100.00	825/825	Beta-lactam resistance

### Phylogenetic analysis of blaOXA-347

**Fig. 1:**
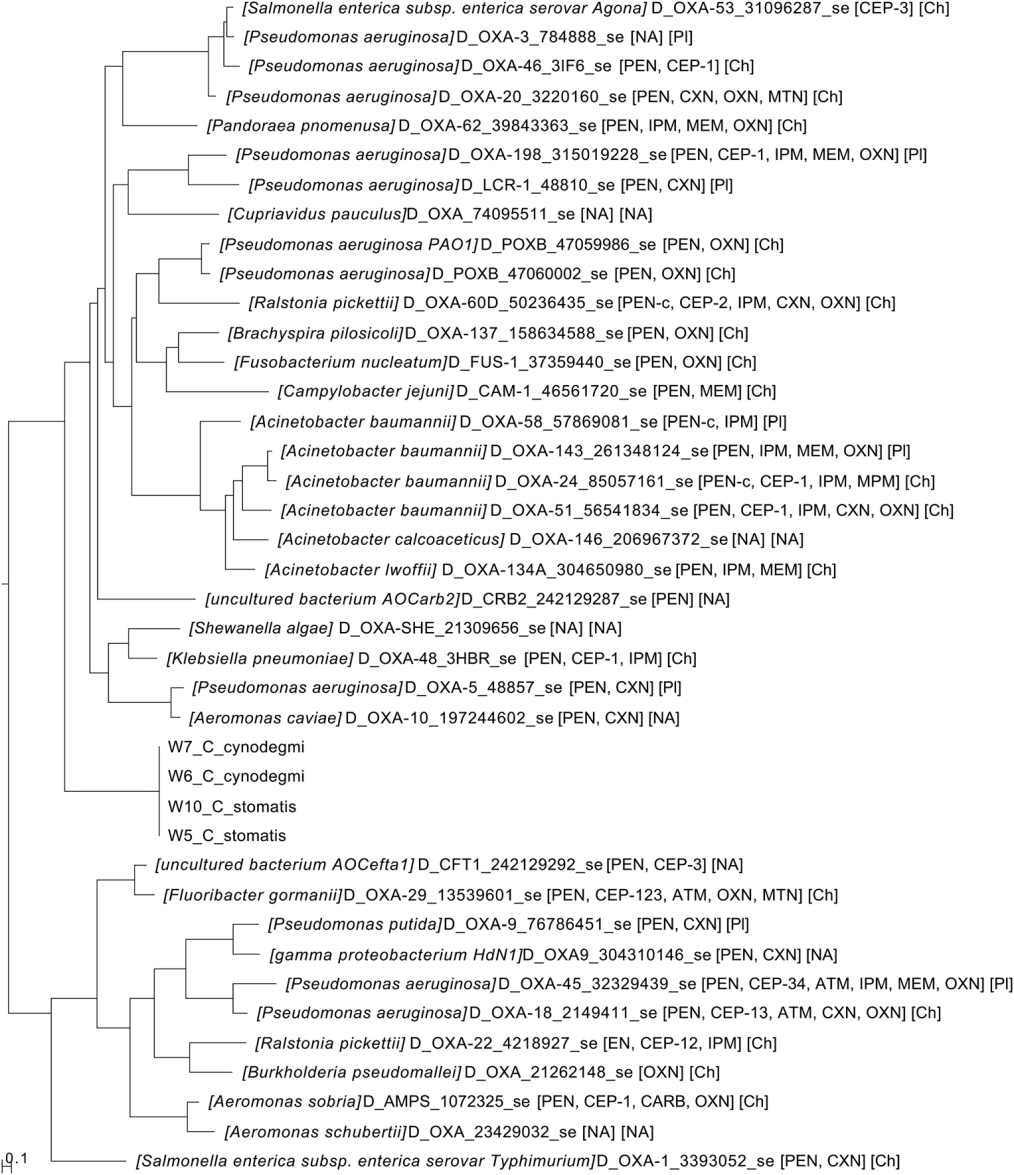
A phylogenetic tree of the beta lactamases showing the relationship between the candidate sequence and other beta lactamases

The class D beta-lactamase gene family is large and diverse [8]. Thus, we set out to analyse the relationship between blaOXA-347 and other class D beta-lactamases utilizing a web-based program [29]. This analysis resulted in a phylogenetic tree, which clearly showed that the *Capnocytophaga* sequences clustered uniformly (100% sequence identity) and were related to several beta-lactamases of clinical importance, including ‘blaOXA48’ [8] (Fig. 1).

## Discussion

Here we set out to describe the antibiotic susceptibility patterns of a collection of animal bite associated *Capnocytophaga* isolates, including the recently discovered *C. canis* and *C. stomatis*. In addition, we linked the phenotypic results with full genome sequences obtained in a previous project [26]. A major finding in this work is the identification of a beta-lactamase in four strains of animal bite associated *Capnocytophaga* species. This enzyme, designated blaOXA347, is associated with phenotypic resistance to penicillin, cephalosporins, and also to imipenem. Given this broad substrate specificity, it could indeed be classified as a carbapenemase [8]. Notably, blaOXA347 was only found in wound-associated strains and not in blood culture isolates, suggesting a possible negative effect on virulence and fitness. The enzyme also appears to be connected to the bacterial chromosome and not found on a plasmid, which significantly lowers the transmission potential to other *Capnocytophaga* strains. However, the fact that the gene is 100% identical at the nucleotide level in the four strains that represent two different species indicates that it has been horizontally transferred.

It is possible that commensal bacteria in the oral flora of cats and dogs serve as a reservoir for antibiotic resistance genes. In humans, for example, *Capnocytophaga* species (*C. ochracea, C. sputigena*) are often resistant to penicillin and cephalosporins by virtue of several different beta-lactamases [6, 10]. In line with this analogy, it is possible that blaOXA347 could be transmitted via transposons to the more virulent and invasive *C. canimorsus*, although no such multidrug resistant *C. canimorsus* have been described. Notably, this mechanism of transmission was recently shown for the chromosomally encoded blaNDM-1 carbapenemase gene in *Acinetobacter baumanii* [14] and OXA48 carbapenemase in highly virulent *E. coli* [2].

Interestingly, a BLAST search revealed that blaOXA347 has a 100% homology to a beta-lactamase in *Myroides odoratimimus* (data not shown). This is an environmental gram-negative bacteria found in soil and water, which in rare cases may cause infections in immunocompromised individuals [7]. *M. odoratimimus* is commonly resistant to beta-lactams, including cephalosporins and carbapenems [16]. Several chromosomally encoded beta-lactamases have been described in this bacterial species [1], and it is possible that it has transmitted blaOXA347 to bacteria in the dog oral flora, although this remains to be proven.

In addition, a neighbour in the phylogenetic tree is *Schewanella algae*, a non-fermentative, gram negative opportunistic bacteria, which may cause skin and soft-tissue infections in immunocompromised individuals. *Schewanella algae* has also been shown to be resistant to cefalosporins and to imipenem [24]. In addition, whole genome sequencing has demonstrated the presence of several beta-lactamases in the *S. algae* genome [5]. Thus, it is possible that the environmental bacteria *M. odoratimimus* and *S. algae* may have transmitted blaOXA-347 to *C. cynodegmi* and *C. stomatis* in the oral flora of cats and dogs.

This study has several strengths. First, it is the most updated and largest analysis of antibiotic susceptibility in animal bite associated *Capnocytophaga* species in more than 25 years [3, 25]. Second, we have included the recently discovered novel species *C. canis* and *C. stomatis*, for which there is no data on antibiotic susceptibility available. Notably, blaOXA347 was found in two isolates of *C. stomatis*, which make this species a focus for future studies in the area of bacterial resistance. Finally, we have combined detailed phenotypic testing with whole genome analysis, which has not previously been performed for *Capnocytophaga* species.

There are also several weak points. Although the study includes 24 isolates (whole genomes) and 16 isolates (phenotypic analysis), it is a small study compared to studies on many other bacterial species. This has to be taken into account when interpreting the data, and the full picture of the epidemiology of blaOXA347 in animal bite associated *Capnocytophaga* species remains to be shown. Another limitation includes the bioinformatic analysis, which was performed utilizing the web-based program ResFinder. Interestingly, ResFinder rapidly identified blaOXA347 and tet(X) in several isolates. However, the phenotypic antibiotic resistance patterns indicated that there are several additional resistance genes encoded in the genomes of these bacteria. Taken together, this discrepancy indicates that more detailed bioinformatic work needs to be done to describe the full resistome on the genome level. Until efficient and easy-to-use bioinformatics tools are readily available for clinicians, phenotypic testing will still have a place in the clinical laboratory.

It is noteworthy that all *C. canimorsus* strains tested had a very low MIC value for penicillin, the first-line drug for infections after cat or dog-bites. This finding is in line with previous studies [3, 25]. Based on our data (Table 1), penicillin or cephalosporins or in the case of type I allergy to PC, clindamycin or tetracycline appear to be effective against invasive infections with *C. canimorsus*. For wound infections, the situation is more complex, and there is broad resistance to beta-lactams in four isolates. However, no resistance to clindamycin was found, and tetracycline appears to be a good alternative. Thus, we suggest that clinicians become aware of the possibility of beta-lactam resistance in wound-associated *Capnocytophaga* species, and that full AST is performed in case of animal bite associated wounds. Likewise, the presence of a beta-lactam resistant *C. canimorsus* is unlikely, but—given the current data—a future possibility, which needs to be considered in clinical bacteriological laboratories. This is particularly important, since *Capnocytophaga* are fastidious bacteria that grow slowly and can be difficult to identify, especially in wound specimens where a polymicrobial flora dominates.

To conclude, we demonstrated the antibiotic susceptibility profile of clinical *Capnocytophaga* isolates, and have identified a chromosomally encoded betalactamase (blaOXA347) with broad substrate specificity in animal associated *Capnocytophaga* species. This is a novel finding, and points to a role of the normal flora of pet animals as a reservoir of bacterial resistance genes, although the clinical relevance remains to be established. We suggest that further research is warranted in order to obtain more knowledge on the epidemiology, bacterial resistance, and clinical outcomes of *Capnocytophaga* infections.
